# Common Prognostic Biomarkers and Outcomes in Patients with COVID-19 Infection in Saudi Arabia

**DOI:** 10.3390/tropicalmed8050260

**Published:** 2023-04-30

**Authors:** Mashael Abujabal, Mohamed A. Shalaby, Layla Abdullah, Amr S. Albanna, Mohamed Elzoghby, Ghadeer Ghazi Alahmadi, Sidharth Kumar Sethi, Mohamad-Hani Temsah, Fadi Aljamaan, Khalid Alhasan, Jameela A. Kari

**Affiliations:** 1 Pediatric Nephrology Unit, Faculty of Medicine and Pediatric Nephrology Center of Excellence, King Abdulaziz University Hospital, King Abdulaziz University, Jeddah 21589, Saudi Arabia; 2 King Abdullah International Medical Research Center, King Saud Bin Abdulaziz University for Health Sciences, Jeddah 14611, Saudi Arabia; 3 Pediatric Intensive Care Unit, Pediatrics Department, College of Medicine, King Abdulaziz University Hospital, Jeddah 21589, Saudi Arabia; 4 Department of Pediatric, College of Medicine, King Abdulaziz University, Jeddah 21589, Saudi Arabia; 5 Kidney and Renal Transplant Institute, Medanta, The Medicity Hospital, Gurgaon 122001, India; 6 Pediatric Department, College of Medicine, King Saud University, Riyadh 11451, Saudi Arabia; 7 Prince Abdullah bin Khaled Coeliac Disease Research Chair, King Saud University, Riyadh 11362, Saudi Arabia; 8 Critical Care Department, College of Medicine, King Saud University, Riyadh 11451, Saudi Arabia; 9 Department of Kidney and Pancreas Transplantation, Solid Organ Transplant Center of Excellence, King Faisal Specialist Hospital and Research Center, Riyadh 12713, Saudi Arabia

**Keywords:** biomarkers, COVID-19, common prognostic biomarkers

## Abstract

**Background:** COVID-19 is a respiratory disease that eventually became a pandemic, with 300 million people infected around the world. Alongside the improvement in COVID-19 management and vaccine development, identifying biomarkers for COVID-19 has recently been reported to help in early prediction and managing severe cases, which might improve outcomes. Our study aimed to find out if there is any correlation between clinical severity and elevated hematological and biochemical markers in COVID-19 patients and its effect on the outcome. **Methods:** We have collected retrospective data on socio-demographics, medical history, biomarkers, and disease outcomes from five hospitals and health institutions in the Kingdom of Saudi Arabia. **Results:** Pneumonia was the most common presentation of COVID-19 in our cohort. The presence of abnormal inflammatory biomarkers (D-dimer, CRP, troponin, LDH, ferritin, and t white blood cells) was significantly associated with unstable COVID-19 disease. In addition, patients with evidence of severe respiratory disease, particularly those who required mechanical ventilation, had higher biomarkers when compared to those with stable respiratory conditions (*p* < 0.001). **Conclusion:** Identifying biomarkers predicts outcomes for COVID-19 patients and may significantly help in their management.

## 1. Introduction

COVID-19 is a type of respiratory disease that rapidly became a pandemic around the world [1]. It triggered a great effect across the world in several aspects, such as the implementation of lockdowns, social distancing, and travel restrictions. While the majority of infected people have mild symptoms, around 20% of all COVID-19 patients experience severe symptoms that include hemodynamic instability, respiratory failure, hypoxia, and failure of multiple organs [2]. Ventilation equipment and oxygenation supports are often required for severe cases [3].

It is reported that around 30% of the total severe cases eventually lead to death, as compared to low mortality in those with mild symptoms [1]. It was also noted that the genetic diversity and extreme recombination rates of the different coronaviruses known (SCoV, MERS_CoV, and SCoV2) imply that more outbreaks will possibly occur, leading to diverse outcomes in patients [4]. Patients that had serious COVID-19 experienced pneumonia on a computerized tomography (CT) scan, ground-glass opacity, pneumonitis, and lung damage [5,6]. Multisystem inflammatory syndrome (MIS) is a type of extra-pulmonary complication more commonly present in the younger population that acquired COVID-19 [7,8]. Previous reports have described MIS laboratory features in children (MIS-C) that have a relationship to the known hyperinflammatory syndrome [9].

Clinical assessments of COVID-19 patients must be performed to identify those that are at risk. Laboratory biomarkers could present information that significantly affects patient care quality.

Biomarkers are quantifiable biochemical substances utilized for recognizing and indicating an ailment’s presence and severity [10,11]. Using these biomarkers in a clinical setting could present information that significantly affects patient care quality. These markers could be used in the early recognition of severe cases and identification of those who are at-risk, which may serve as clinical predictors of severe and fatal COVID-19 [12]. On the other hand, they can also be sorted into categories such as (1) immunological and inflammatory host immune response markers, (2) hematological abnormality markers, and (3) end-organ injury and systemic response markers [13]. One type of biomarker is a molecular biomarker in the form of genes, proteins, glycans, or metabolites that are utilized for the evaluation of a disorder and its treatment and contribute to the expansion of biomedical development [1]. 

It was reported that there were changes in hematology and biochemical parameters, including lymphocyte count, neutrophil count, C-reactive protein (CRP), and erythrocyte sedimentation rate (ESR). While there were also findings of high interleukin 6 (IL-6), lactate dehydrogenase (LDH), high blood sugar, and gamma-glutamyl transferase (GGT) in more severe COVID-19 patients [14,15,16,17,18].

A study performed by Velichko et al. provides a fast, reliable, and cost-effective alternative mobile tool for the diagnosis of COVID-19 based on the routine blood values (RBVs) measured at the time of admission. In this study, 13 popular classifier machine learning models and the LogNNet neural network model were run on 51 routine blood values to detect patients infected with COVID-19. The histogram-based gradient boosting (HGB) model was the most successful classification model in terms of accuracy and time in detecting the diagnosis of the disease [19]. 

Our study aimed to investigate the correlation between clinical severity and elevated hematological and biochemical markers in COVID-19 patients and also determine if these changes could predict the outcome. This might guide clinicians to identify severe cases and provide early and appropriate management.

## 2. Materials and Methods

### 2.1. Study Design and Setting

The current research was designed to study common inflammatory biomarkers and their relationship to outcomes in patients with COVID-19 infection. This retrospective cohort multicenter study was carried out in five hospitals and health institutes in the Kingdom of Saudi Arabia (King Abdulaziz University Hospital, King Saud University Medical City, East Jeddah Hospital, King Fahd Medical City, and Prince Mohammed bin Abdulaziz Hospital). All these hospitals and centers are qualified to receive and treat COVID-19 patients.

### 2.2. Subjects and Methods

We have revised the data of all patients admitted to different units of participating hospitals (emergency rooms, medical wards (adult and pediatric), and different intensive care units) over the period of March 2020 to July 2020, before the COVID-19 vaccination era. This period represented the peak of the COVID-19 epidemic wave in Saudi Arabia. The COVID-19 infection was confirmed using a real-time reverse transcription polymerase chain reaction (RT-qPCR) test for the qualitative detection of nucleic acid from SARS-CoV-2 from respiratory tract specimens (nasopharyngeal swabs or endotracheal aspiration).

### 2.3. Inclusion Criteria

All positive patients, including all age groups, with either initial positive swabs or those initially negative but turned positive with subsequent repeated swabs were included.

### 2.4. Exclusion Criteria

Patients with any medical conditions or receiving any medication that might affect the level of tested markers and those with insufficient data were excluded. For example, patients recorded with a history of any disease that would have a significant influence on the coagulation profile were excluded, as were those with a history of ischemic heart disease, liver disease, alcoholism, heparin, aspirin, and warfarin long-term medication use, current drug effects on coagulation, and platelet count.

We have collected baseline, demographic, and clinical data of all recruited patients (adult and pediatric population): gender, age, body mass index, and associated comorbidities (diabetes mellitus, hypertension, renal disease, cardiac disease, respiratory disease, hematology, and oncology diseases, post-transplant, and central nervous system disorders) (Table 1). The presenting symptoms and signs are listed in Table 2.

The following laboratory findings of all patients were revised: hemoglobin (HGB), white blood cell (WBC) counts, platelet counts, C-reactive protein (CRP), ferritin, fibrinogen, and D-dimer. In the view of a retrospective study, the timing of taking blood samples was determined by the treating physician according to clinical conditions. We collect available data on the mentioned blood values, and we add the percentage of valid data for each to Table 3 and Table 4. We used AUC—ROC curve performance measurement to represent the degree or measure of separability and to know how much the lab results are capable of distinguishing unstable cases and inflammatory biomarkers [20]. All data on AUC, sensitivity, specificity, accuracy, and confidence intervals of biomarkers are shown in Table 4. We select the most commonly used inflammatory markers from inflammatory and hematology subtypes.

### 2.5. Definitions

Hypoxia was defined as a state of decreased oxygen delivery in sufficient amounts at the tissue level to maintain adequate homeostasis, guided by clinical and laboratory parameters such as decreased oxygen saturation, increasing oxygen requirement, and evidence of respiratory failure.

Pneumonia is defined as clinical or radiological signs of pneumonia.

Unstable COVID-19 indicates the requirement of invasive mechanical ventilation, vasopressors, inotropic support, or cardiopulmonary arrest.

The KDIGO definition was used to define acute kidney injury as an increase in serum creatinine level to more than 0.3 mg\dl (26.5 mmol\L). Within 48 h., there was an increase in serum creatine of more than 1.5 times of baseline, and a reduction in urine output to less than 0.5 mL\kg\hr [21].

We look at abnormal levels of biomarkers as an indicator of the severity of COVID-19, not as diagnostic purposes for COVID-19, for abnormally low levels of any laboratory or inflammatory markers, referred to as the lowest blood level measured during admission, in comparison with the reference of institutional labs. In the same way, an abnormally high level of any laboratory or inflammatory marker refers to the highest blood level measured during admission in comparison with the reference level of institutional laboratories. All cutoff levels are listed in Table 3.

The protocol was revised and approved by the ethical committee at King Abdulaziz University Hospital (Reference Number: 510-22). The privacy and confidentiality of the patients were strictly observed. All the collected data were kept in a secure place.

No conflict of interest was associated with this study. 

### 2.6. Statistical Analysis

All analyses were performed using STATA (StataCorp. 2011. Stata Statistical Software: Release 12. College Station, TX: StataCorp LP) software. The proportion and mean/median for dichotomous and continuous variables, respectively, were measured to describe patients’ characteristics. The proportion and 95% confidence interval (CI) of different signs and symptoms among all included subjects were estimated. The proportion and 95% CI of patients with abnormal blood levels of different biomarkers were compared between stable and unstable COVID-19 patients using the chi-square test. In addition, the mean value of these biomarkers was measured in both groups (the comparison was based on estimating the difference in frequency of abnormal blood levels between groups, rather than the mean blood level, as it is more relevant clinically). The proportion of abnormal inflammatory markers was compared between COVID-19 patients with different disease severity using a chi-square test. The receiver operating curve (ROC) was used to compare the performance characteristics of inflammatory biomarkers for the diagnosis of severely unstable COVID-19 patients. *p* < 0.05 was considered statistically significant, and *p* values were calculated at 95% CI levels.

## 3. Results

**Figure 1:**
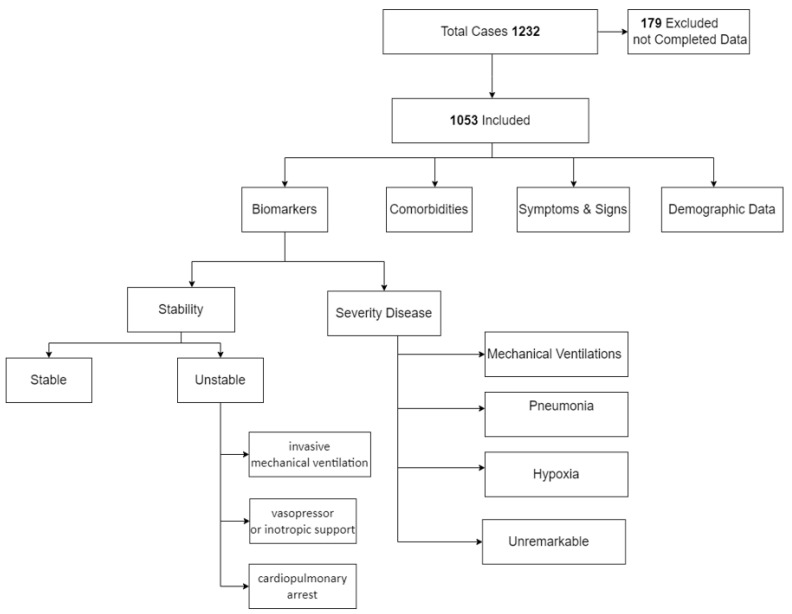
Study flowchart.

We reviewed the data of 1232 patients admitted with COVID-19 positivity. We excluded 179 patients due to either insufficient data or because subjects had medical conditions or were receiving medications that might compromise the level of biological markers. The study recruited 1053 subjects (64.6% were males) (Figure 1). The socio-demographic characteristics of the included subjects in the study are listed in Table 1.

More than half of our cohort (57.5%) had at least one comorbidity. Diabetes mellitus was the most common comorbidity (36%), followed by hypertension (32.7%), cardiac disease (14.6%), respiratory disease (11.6%), and renal disease (8.7%). 

**Figure 2:**
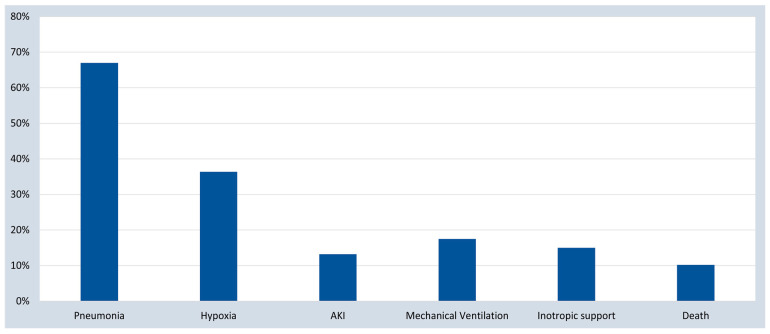
COVID-19 disease severity and outcomes. AKI, acute kidney injury.

The presenting signs and symptoms of the studied patients are shown in Table 2. The majority (93.3%) of the included patients were symptomatic. The most common reported symptom was fever (72.5%, 69.6–75.0), followed by respiratory symptoms (cough (66.7%, 63.7–69.5), dyspnea (56.4%, 53.4–59.4), and flu-like symptoms in almost 14%). Gastrointestinal symptoms were reported in 20.1%, mainly in the younger age group. Similarly, fever was observed as the commonest clinical sign in more than half of the studied subjects (53.9%, 50.9–57.0), followed by one-third having mild respiratory distress (33.9%, 31.0–36.9), and nearly one-fourth having severe respiratory distress (19.6%, 17.2–22.1). On the other hand, we looked at severe organ involvement and failure as an indicator of COVID-19 disease severity and outcomes (Figure 2). Results revealed that pneumonia was the most prominent disease manifestation, followed by hypoxia, mechanical ventilation, inotropic support, acute kidney injury, and death.

**Figure 3:**
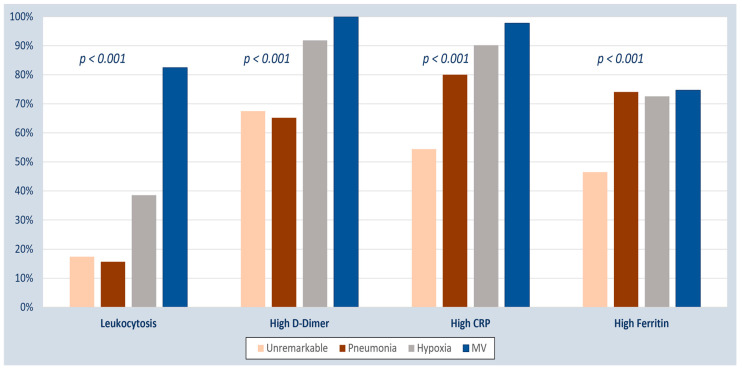
Inflammatory biomarkers among COVID-19 patients with different disease severity, categorized based on respiratory involvement. CRP, C-reactive protein, MV, mechanical ventilation.

The presence of abnormal inflammatory biomarkers was significantly associated with unstable COVID-19 disease (Table 3). The mean biomarker level among patients with stable and unstable COVID-19 disease is shown in Table 4. Patients with worse respiratory illnesses, especially those who required mechanical ventilation, were more likely to have abnormal inflammatory biomarkers (Figure 3).

**Figure 4:**
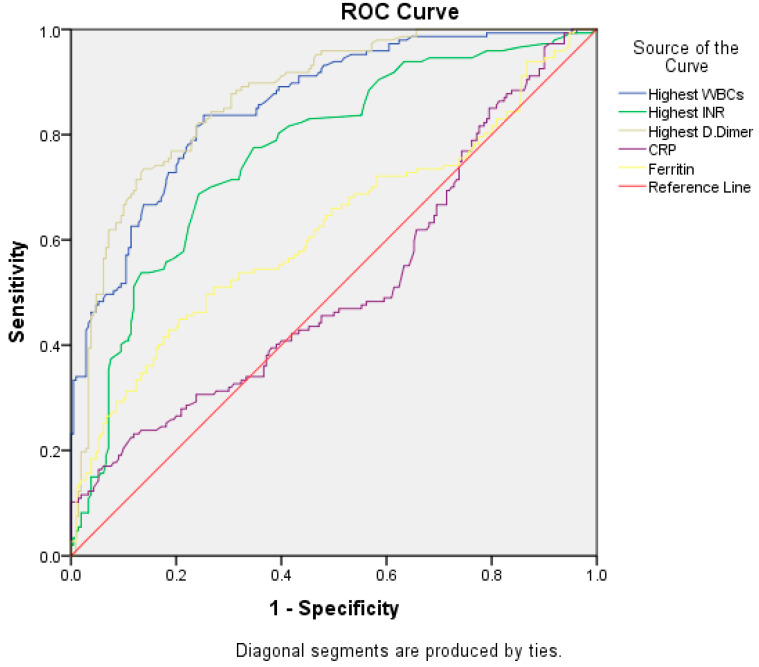
ROC Curve.

The ROC curve (Figure 4) shows that high WBC counts and a high D-dimer are more sensitive predictive markers than CRP in the diagnosis of unstable severe COVID-19. (The area under the curve is 0.85 and 0.87, respectively, with a *p*-value less than 0.001. While for CRP, the area under the curve is 0.61 with a *p*-value of 0.64).

Table 5 shows the diagnostic performance of the most commonly used biomarkers, depending on the ROC curve analysis. Leukocytosis and a high D-dimer show good diagnostic performance; however, a high INR and elevated CRP performance were fair, and an elevated ferritin level was poor.

## 4. Discussion

In this multicentric study with 1053 subjects, we found that most hospitalized patients due to COVID-19 are male (64.6% of patients). This is similar to previous studies; Guan et al. reported that 58.1% of affected patients were male [22]. The risk of death was also reported in many studies to be higher in male patients [23,24,25,26], which might indicate biological risk determinants as an important factor in COVID-19 disease severity.

In our study, we found that 57.5% of all patients had at least one comorbidity, with diabetes being the most common. This is similar to several previous studies; a metanalysis of 48 studies revealed that diabetes, hypertension, cardiovascular disease, renal disease, and malignancies are associated with a high incidence of death in COVID-19 patients [27]. 

Fever and respiratory symptoms were very common in our cohort. In a previous retrospective study, we looked at risk factors related to COVID-19 death among 229 critically ill patients in five hospitals in KSA. We have found that signs and symptoms presented by hospitalized patients who had died did not substantially differ when compared to those who survived. Kidney disease, cardiac disease, and diabetes were substantially correlated with in-hospital mortality [28]. Another retrospective study of 439 COVID-infected subjects from a single center in Riyadh observed associated comorbidities such as diabetes, vitamin D deficiency, hypertension, and obesity. While a higher death rate was observed among diabetic patients as compared to non-diabetic patients. In addition, smoking, old age, β-blocker use, congestive heart failure, higher creatinine, the occurrence of bilateral lung infiltrates, and severe vitamin D deficiency were deemed to be more substantial predictors of worse outcomes. [29].

Biomarkers were correlated with worse COVID-19 disease outcomes. This is online, along with previous reports. A systematic review of 52 articles involving 6320 COVID-19-positive patients revealed that elevated levels of neutrophil count, WBC, prolonged PT, ESR, D-dimer, fibrinogen, procalcitonin, IL-6, and IL-10 can more significantly progress to a more serious form of COVID-19 infection. Similarly, elevated D-dimer, neutrophils, WBCs, and prolonged PT are substantially associated with intensive care admission. On the other hand, mortality was linked with high CRP, IL-6, neutrophil, and D-dimer levels [30].

Meta-analysis research involving 18 studies recommends clinicians closely monitor WBC count, lymphocyte count, platelet count, IL-6, and serum ferritin as markers for potential progression to critical illness [12]. Another systematic review involving 4848 COVID-positive patients from 23 studies suggested that severe COVID-infected patients have elevated CRP, procalcitonin, LDH, and D-dimer but lower levels of albumin than those with non-severe COVID-19 infections [31]. Additionally, we have reported previously that elevated APTT, INR, ferritin, and acidosis were the independent factors that influenced mortality among critically ill COVID-infected participants [28]. Huyut et al. showed that the most important rapid blood values in the diagnosis of the COVID-19 disease were MCHC, MCH, and Aptt, and the most important rapid blood values in the prognosis of the disease were ESR, NEU, CRP, albumin, and RBC [24]. In addition, cardiac biomarkers such as N-terminal pro-B-type natriuretic peptide (NT-proBNP) and notably high-sensitive cardiac troponin (hs-cTn) can also be considered key prognostic predictors among COVID-19-infected patients [32]. Meta-analysis research involving 17,794 COVID-19-infected subjects revealed that adverse outcomes were developed in patients with elevated AST and cardiac troponin I levels [33]. This might be consistent to some extent with our study, where we found that a high level of troponin significantly correlated with hemodynamic instability in COVID-19 patients.

Furthermore, a systematic review involving 1735 patients from 21 studies characterized inflammatory markers to compare non-severe/severe COVID-19 against MIS-C, non-severe against severe MIS-C, and MIS-C across different age ranges. Patients with MIS-C had reduced absolute lymphocyte count (ALC) and elevated absolute neutrophil count (ANC), CRP, and D-dimer levels when compared with non-severe COVID-19-infected patients. Ponti et al. stated that the CRP marker was found to be significantly increased in the initial phases of the infection for severe COVID-19 patients, also before indications of critical findings with CT [34]. Lippi et al. reviewed 217 articles, and only 11 met inclusion criteria, demonstrating that many laboratory abnormalities were found in patients with unfavorable progression of coronavirus disease 2019 [35]. In addition, CRP has been associated with disease development and is an early predictor for severe COVID-19. On the other hand, patients with MIS-C had elevated ESR as well as reduced platelet count (PLT) and LDH when compared with severe COVID-19-infected patients. Patients with non-severe MIS-C had substantially reduced levels of CRP, ANC, WBC, ferritin, and D-dimer than patients with severe forms of MIS-C. Adolescents and older children with MIS-C were found to have elevated ferritin and CRP as compared to children aged between 0 and 5 years old with the multisystem inflammatory syndrome in children (MIS-C) [9]; however, not one of our patients presented with the clinical criteria of MIS-C. A study involving 6026 COVID-19-infected patients from Hafar Al-Batin and Riyadh cities, with a mortality rate of 23%, revealed that mortality is significantly influenced by older age as well as increased neutrophil and WBC counts and D-dimer levels [36].

We have observed that high LDH, alanine aminotransferase, and aspartate aminotransferase are associated with severe and unstable COVID-19 patients. Alguwaihes et al. reported that elevated blood glucose and creatinine levels, increased alanine aminotransferase, and neutrophil count were significantly associated with ICU admission [29]. Similarly, Beairwa et al. stated that lowered LDH, alanine aminotransferase, and aspartate aminotransferase levels have been observed to have a substantial correlation with COVID-19-infected patient mortality [14], and this could be explained by that liver affection, which might happen as part of multiorgan system failure in severe cases.

Our study shows the distribution of inflammatory biomarkers among COVID-19 patients with various severity levels, with a significant correlation between the inflammatory biomarkers and the patient’s disease severity. A higher number of patients who required mechanical ventilation because of disease severity had abnormal biomarkers such as leukocytes, high D-dimer, high CRP, and high ferritin. A previous study of 352 critically ill COVID-19-infected patients in Riyadh revealed that about 56.8% were mechanically-ventilated patients upon ICU admission, having a peripheral oxygen saturation/fraction of inspired oxygen (SpO2/FiO2) ratio of 158 ± 32. A decreased SpO2/FiO2 ratio, active smoking, old age, pulmonary embolism, and heightened D-dimers and lactate were deemed significant predictors of mortality [37].

The ROC curve shows that the highest WBC and high D-dimer are more sensitive to diagnosing severe unstable COVID-19 with good diagnostic performance than CRP and high ferritin with fair to poor diagnostic performance (Table 5). Elkhalalifa et al. published data using the ROC curve, which showed that D-dimer is an important marker for patients hospitalized with severe and unstable COVID-19 [30]. Manuela et al. also used the ROC curve and reported that high CRP can predict the worsening of clinical conditions [38].

## 5. Conclusions

Identifying biomarkers for COVID-19 patients may significantly help in managing the affected patients, especially the immune-compromised population. In addition, these biomarkers could be of valuable help in screening patients with varying severity of COVID-19 infection.

### Limitations of Study

We think that more quantitative studies on the threshold levels of these biomarkers are required to continuously monitor disease progression. As such, dynamic monitoring of these biomarkers as well as a longer follow-up duration are recommended for further understanding of the COVID-19 prognosis. These data were collected before the COVID-19 vaccination era, which might explain the high percentage of severe cases.

Also, more studies are required to compare the levels of the biomarkers in cases of non-COVID-19 pneumonia.

## Figures and Tables

**Table 1:** Baseline patients’ demographic and disease characteristics.

Characteristics	Estimate
**Age in years./mean (SD)**	46.2 (19.5)
**Agein years/median (IQR)**	48 (34–60)
**Male Sex**	680 (64.6)
**BMI—mean (SD)**	28.1 (11.3)
**BMI—median (IQR)**	27.0 (23.7–30.9)
**Comorbidities—No. (%)**	
Diabetes	379 (36.0)
Hypertension	344 (32.7)
Renal disease	92 (8.7)
Cardiac disease	154 (14.6)
Respiratory disease	122 (11.6)
Hematology disorder	31 (3.0)
Oncology disorder	30 (2.9)
Post-transplant	6 (0.6)
CNS disorder	4 (1.8)
HIV infection	2 (0.9)
Any comorbidity	605 (57.5)

BMI, body mass index; CNS, central nervus system; HIV, human immunodeficiency virus.

**Table 2:** Presenting signs and symptoms.

Characteristics	% (95% CI)
**Symptoms**	
Fever	72.4 (69.6–75.0)
Cough	66.7 (63.7–69.5)
Dyspnea	56.4 (53.4–59.4)
Diarrhea	20.1 (17.7–22.7)
Headache	6.2 (4.8–7.8)
Muscle Pain	5.2 (4.0–6.7)
Skin rash	0.9 (0.4–1.6)
Flu-like symptoms	14.3 (12.3–16.6)
Any Symptom	93.3 (91.6–94.7)
**Signs**	
Fever	53.9 (50.9–57.0)
Mild respiratory distress	33.9 (31.0–36.9)
Severe respiratory distress	19.6 (17.2–22.1)
Heart failure	2.6 (1.7–3.7)
Hematemesis	0.6 (0.2–1.2)
Dehydration	3.5 (2.5–4.8)
Tonsilitis	5.9 (4.5–7.5)
No clinical signs	14.7 (12.6–17.0)

**Table 3:** Proportion of patients with abnormal biomarkers among patients with stable and unstable COVID-19 disease.

Biomarkers	Stable		Unstable		
	Estimate	95% CI	Estimate	95% CI	*p*-Value
Anemia (%)	32.3	29.0–35.8	57.3	50.4–64.1	<0.001
Leukocytosis (%)	20.2	17.3–23.4	77.0	70.3–82.8	<0.001
Leukopenia (%)	29.5	26.3–32.9	20.6	15.3–26.7	0.011
Thrombocytopenia (%)	12.7	10.2–15.6	41.6	34.7–48.7	<0.001
High PT (%)	83.0	79.6–86.1	95.2	91.3–97.7	<0.001
High APTT (%)	46.3	42.0–50.7	79.9	73.8–85.1	<0.001
High INR (%)	8.3	6.1–10.9	50.0	43.1–56.9	<0.001
High fibrinogen (%)	14.5	10.4–19.5	92.3	86.9–95.9	<0.001
High D-dimer (%)	67.7	62.4–72.6	99.0	96.3–99.9	<0.001
High CRP (%)	72.3	68.7–75.7	96.3	92.8–98.4	<0.001
High troponin I	49.7	44.3– 55.2	81.9	75.2–87.5	<0.001
High ferritin (%)	62.2	58.2–66.1	75.9	69.2–81.9	0.001
High LDH (%)	73.5	69.5–77.2	97.5	94.2–99.2	<0.001
Disturbed LFT (%)	47.0	43.4–50.6	84.7	79.3–89.2	<0.001
AKI (%)	1.4	0.7–2.5	56.6	49.9–63.2	<0.001

WBC, white blood count; PT, prothrombin time; APTT, partial thromboplastin time; INR, international normalized ratio; CRP, C reactive protein; LDH, lactate dehydrogenase.

**Table 4:** Mean biomarker level among patients with stable and unstable COVID-19 disease.

Biomarkers	Stable		Unstable *	
	Estimate	95% CI	Estimate	95% CI
Hemoglobin (mean/SD) ^$^	12.7/2.1	12.5–12.8	10.9/3.2	10.4–11.3
Highest WBC (mean/SD) ^@^	8.8/4.1	8.5–9.1	18.0/9.1	16.6–19.3
Lowest WBC (mean/SD) ^$^	6.0/2.8	5.8–6.2	7.4/3.8	6.8–7.9
Platelet (mean/SD) ^$^	261/115	251–270	205/145	184–224
PT (mean/SD) ^@^	13.8/2.2	13.7–14.0	19.4/17.3	17.3–21.4
APTT (mean/SD) ^@^	37.9/14.1	36.7–39.2	76.9/51.9	69.8–84.0
INR (mean/SD) ^@^	1.11/0.2	1.09–1.13	1.54/1.1	1.39–1.69
Fibrinogen (mean/SD) ^@^	81/205	55–106	674/656	569–778
D-dimer (mean/SD) ^@^	2.4/8.1	1.5–3.3	16.3/20.6	13.3–19.2
CRP (mean/SD) ^@^	32.3/47	28.7–35.9	82.1/101	68.6–95.6
Troponin I (mean/SD) ^@^	5.3/13.3	3.9–7.0	3.0/8.9	1.7–4.4
Ferritin (mean/SD) ^@^	583/965	506–659	1409/1850	1142–1675
LDH (mean/SD) ^@^	383/213	365–402	931/909	804–1057
ALT (mean/SD) ^@^	55/57	51–59	218/548	145–290
AST (mean/SD) ^@^	51/55	47–55	235/601	153–316
Creatinine (mean/SD) ^@^	96/133	86–105	348/384	297–399
GFR (mean/SD) ^$^	84/35	82–87	47/39	41–52

WBC, white blood count; PT, prothrombin time; APTT, partial thromboplastin time; INR, international normalized ratio; CRP, C reactive protein; LDH, lactate dehydrogenase; ALT, alanine transaminase; AST, aspartate transaminase; LFT, liver function test; GFR, glomerular filtration rate. * Unstable conditions indicate a requirement for invasive mechanical ventilation, vasopressors, inotropic support, or cardiopulmonary arrest. ^$^ Lowest blood level measured during admission. ^@^ Highest blood level measured during admission.

**Table 5:** ROC curve results of different biomarkers with diagnostic performance.

Test Result Variable(s)	Area (AUC)	Diagnostic Performance	*p*-Value	Cut off Point	Sensitivity	Specificity	Accuracy	95% Confidence Interval	
								Lower Bound	Upper Bound
Highest WBCs k/uL	0.853	Good (B)	<0.001	12.8500	0.752	0.735	0.738	0.807	0.899
Highest INR (ratio)	0.777	Fair (C)	<0.001	1.21500	0.664	0.571	0.591	0.718	0.836
Highest D-dimer mg/L	0.872	Good (B)	<0.001	3.26800	0.721	0.378	0.454	0.827	0.917
CRP mg/L	0.531	Fail (F)	0.410	16.9000	0.650	0.483	0.520	0.457	0.604
Ferritin ng/L	0.653	Poor (D)	<0.001	170.550	0.801	0.326	0.431	0.583	0.723

WBC, white blood count; PT, prothrombin time; INR, international normalized ratio; CRP, C reactive protein; AUC, Area Under Curve.

## Data Availability

Data are available on request.
